# Proteomic analyses reveal differences in cold acclimation mechanisms in freezing-tolerant and freezing-sensitive cultivars of alfalfa

**DOI:** 10.3389/fpls.2015.00105

**Published:** 2015-02-27

**Authors:** Jing Chen, Guiqing Han, Chen Shang, Jikai Li, Hailing Zhang, Fengqi Liu, Jianli Wang, Huiying Liu, Yuexue Zhang

**Affiliations:** ^1^College of Life Sciences and Technology, Harbin Normal UniversityHarbin, China; ^2^Institute of Grass Research, Heilongjiang Academy of Agricultural SciencesHarbin, China

**Keywords:** proteomics, cold acclimation, RuBisCO, metabolism, homeostasis, alfalfa

## Abstract

Cold acclimation in alfalfa (*Medicago sativa L.*) plays a crucial role in cold tolerance to harsh winters. To examine the cold acclimation mechanisms in freezing-tolerant alfalfa (ZD) and freezing-sensitive alfalfa (W5), holoproteins, and low-abundance proteins (after the removal of RuBisCO) from leaves were extracted to analyze differences at the protein level. A total of 84 spots were selected, and 67 spots were identified. Of these, the abundance of 49 spots and 24 spots in ZD and W5, respectively, were altered during adaptation to chilling stress. Proteomic results revealed that proteins involved in photosynthesis, protein metabolism, energy metabolism, stress and redox and other proteins were mobilized in adaptation to chilling stress. In ZD, a greater number of changes were observed in proteins, and autologous metabolism and biosynthesis were slowed in response to chilling stress, thereby reducing consumption, allowing for homeostasis. The capability for protein folding and protein biosynthesis in W5 was enhanced, which allows protection against chilling stress. The ability to perceive low temperatures was more sensitive in freezing-tolerant alfalfa compared to freezing-sensitive alfalfa. This proteomics study provides new insights into the cold acclimation mechanism in alfalfa.

## INTRODUCTION

A lack of cold tolerance is a major limiting factor for crop production, especially in northern areas. Plants exposed to low but non-freezing temperatures have developed strategies for adapting to stress and enhance cold tolerance via many complex physiological and biochemical changes. Such adaptations are primarily attributed to changes in the expression of functional and regulatory genes (Gomat et al., 2011). Numerous studies have demonstrated that cold acclimation is associated with structural and compositional modifications of compatible solutes in various subcellular compartments and changes in the transcriptome and metabolome (Knaupp et al., 2011; Zuther et al., 2012; Vaclavik et al., 2013). Although cold acclimation mechanisms have been studied for many years, the molecular and genetic foundations of this adaptation in plants remain largely unknown.

Proteomics bridges the gap between gene expression and metabolism (Heazlewood, 2011; Carrol et al., 2013), promoting the study of adaptive mechanisms to chilling stress in many plants (Cui et al., 2005; Amme et al., 2006; Yan et al., 2006; Hashimoto and Komatsu, 2007; Degand et al., 2009; Gao et al., 2009; Lee et al., 2009; Balbuena et al., 2011; Rinalducci et al., 2011; Sánchez-Bel et al., 2012a,b; Uváčková et al., 2012; Takahashi et al., 2013). Changes in proteins play a vital role in cold adaptation due to their direct action on metabolism and biosynthesis pathways. Comparative proteomics were recently applied to analyze changes in cold-sensitive proteins in different cold-tolerant cultivars, such as meadow fescue (leaf), pea (leaf and chloroplast), perennial ryegrass (leaf), strawberry (crown), and winter wheat (leaf; Kosmala et al., 2009; Bocian et al., 2011; Dumont et al., 2011; Koehler et al., 2012; Grimaud et al., 2013; Xu et al., 2013). Proteins involved in energy metabolism, photosynthesis, reactive oxygen species (ROS) scavenging, storage, protection from stress, regulation of the cell cycle and plant development in wheat and barley showed differential abundance between stress-tolerant and stress-sensitive genotypes (Kosová et al., 2014). Proteomics can provide new insights into cold acclimation and improve our understanding of the genetic differences underlying cold tolerance in plants.

High-abundance proteins, such as ribulose-1, 5-bisphosphate carboxylase/oxygenase (RuBisCO), can mask low-abundance proteins of interest. High-abundance proteins reduce the dynamic resolution of proteins and affect the analysis of some functional proteins in proteomic studies. In protein extracts from *Arabidopsis* and rice leaf, ∼12 and 35.3% of the spots, respectively, have been identified as RuBisCO and its derivatives (Giavalisco et al., 2005; Yan et al., 2006). RuBisCO accounts for a high percentage in abundance in green leaf tissue due to protein load capacity limitations (Xi et al., 2006); co-migration with RuBisCO also masks neighboring species (Corthalis et al., 2000; Krishnan and Natarajan, 2009). To overcome this problem, a polyethylene glycol (PEG) mediated pre-fractionation method was used to remove RuBisCO; this protocol depletes most RuBisCO and their derivatives (Kim et al., 2001).

As leguminous forage, alfalfa contributes to biological nitrogen fixation and is widely planted throughout the world. In northern climates, fall dormant cultivars have a greater capability to resist harsh winters than non-dormant varieties (Timmons and Salmon, 1932; Sprague and Fuelleman, 1941; Smith et al., 1979). It has been established that freezing-tolerant and freezing-sensitive cultivars of alfalfa share similar cold regulated (COR) gene complements; differences exist in the rate and extent of the expression of these genes in response to cold. COR genes are induced by low temperature and their translation products can mechanistically protect plants against environmental stresses (Seki et al., 2004). Marked differences were found between propagated clones from genotypes by comparing their gene products, which confirmed that variations in molecular changes that occur at low temperature are under some level of genetic control (Castonguay et al., 2006). Castonguay et al. (2006) hypothesized that some of COR genes fail to be expressed in a timely manner in response to environmental cues in freezing-sensitive alfalfa. The ability to perceive and transduce external signals into a series of molecular events leading to physiological responses differs widely among genotypes within a species (Kaur and Gupta, 2005). However, differences in the cold acclimation mechanisms between freezing-tolerant and freezing-sensitive alfalfa remain unknown. In our study, holoproteins and low-abundance proteins were extracted from alfalfa, and changes in protein categories and relative abundances during chilling were analyzed. Differences in the cold acclimation mechanisms in alfalfa would be first discussed at the protein level using proteomics in our study.

## MATERIALS AND METHODS

### PLANT MATERIAL AND CULTURE CONDITIONS

Two alfalfa cultivars with different freezing tolerances were studied: freezing-tolerant cultivar ZhangDong (ZD), which is fall dormant alfalfa (winter resistant cultivar), and freezing-sensitive cultivar WL525HQ (W5), which is non-dormant alfalfa (winter irresistant cultivar). ZD and W5 seeds were germinated under controlled environmental conditions as follows: 280 μmol/m^2^/s; 16 h light/8 h dark; and a day/night temperature regime of 25/20°C. For the cold treatment, 50 days old seedlings were transferred to a cold chamber set to 4°C for 7 days under other conditions as described above.

### PROTEIN PREPARATION

Leaves (1 g) from both cultivars were sampled at 0 (control), 12 h, and 7 days under cold treatment and were immediately frozen in liquid nitrogen.

#### Holoproteins extraction

For holoproteins extraction, frozen samples were pulverized in a pre-cooled mortar with liquid nitrogen. Samples were then suspended in three volumes of pre-cooled 10% (w/v) trichloroacetic acid (TCA)/acetone with 0.07% (v/v) β-mercaptoethanol and kept at –20°C overnight. The homogenate was centrifuged at 40,000 ×*g* for 1 h at 4°C. The supernatant was removed, and the precipitate was resuspended in three volumes of cold acetone containing 0.07% (v/v) β-mercaptoethanol. The mixture was incubated at –20°C for at least 1 h and centrifuged at 40,000 ×*g* for 1 h at 4°C. The aforementioned steps were repeated until the supernatant was colorless. The vacuum-dried pellet was dissolved in 2 ml of a lysis solution containing 7 M urea, 2 M thiourea, 4% (w/v) 3-[(3-Cholamidopropyl) dimethylammonio]-1-propanesulfonate (CHAPS), 40 mM dithiothreitol (DTT), and 1% (v/v) protease inhibitors (PMSF). The sample was shaken at 4°C for more than 1 h. The mixture was centrifuged at 100,000 ×*g* for 1 h at 4°C to remove any solids. The protein concentration was quantified using a 2D-Quant kit, and the protein solution was stored at –80°C.

#### Low-abundance proteins extraction

Low-abundance proteins were extracted with Mg/NP-40 buffer and fractionated by PEG 4000 (PEG4000), as described by Kim et al. (2001) with slight modifications. Frozen leaves were ground in a pre-cooled mortar with liquid nitrogen. Five milliliters Mg/NP-40 buffer containing 0.5 M Tris-HCl (pH 8.3), 2% (v/v) NP-40, 20 mM MgCl_2_, 2% (v/v) β-mercaptoethanol, 1 mM PMSF, 1 mM ethylene diamine tetraacetic acid (EDTA) and 1% (w/v) polyvinylpolypyrrolidone (PVPP) was added, and the sample was ground on ice for 15 min. After centrifugation at 12,000 ×*g* for 15 min at 4°C, a 50% (w/v) PEG4000 stock solution was added to the supernatant to a final PEG concentration of 17.5% (w/v). The mixture was incubated on ice for 30 min and centrifuged at 12,000 ×*g* for 15 min at 4°C. Three volumes of pre-cooled 10% (w/v) TCA/acetone was added to the supernatant and kept at –20°C overnight. The remaining steps were performed according to the holoproteins extraction method described above.

The quantified holoprotein and low-abundance protein samples were loaded into an SDS-PAGE gel [12.5% (w/v)] and run for ∼2.5 h. The gel was used to determine the quality of proteins and remove RuBisCO.

### TWO-DIMENSIONAL GEL ELECTROPHORESIS

Quantified protein solutions were diluted in rehydration buffer containing 8 M urea, 2% (w/v) CHAPS, 0.3% (w/v) DTT, and 1% (v/v) IPG buffer. IEF strips (24 cm, linear pH 4–7) were rehydrated in 450 μl of rehydration protein solution at 20°C for 12 h in an Ettan IPGphor 3 electrophoresis system (GE Healthcare). IEF was run using the following parameters: 200 V for 2 h, 500 V for 2 h, 1000 V for 2 h, 8000 V for 3 h, and 8000 V for 65000 VH. After IEF, the strips were equilibrated twice in equilibration buffer [6 M urea, 1.5 M Tris-HCl (pH 8.8), 30% (v/v) glycerol, and 2% (w/v) SDS] with 1% (w/v) DTT for 15 min and then with 4% (w/v) iodoacetamide for 15 min. For two-dimensional electrophoresis, the strips were placed on a 12.5% (w/v) SDS-PAGE gel and run at 1 w per gel for 1 h, followed by 13 w per gel for 4.5–5 h. An Ettan DALTSix system (GE Healthcare) was used for two-dimensional electrophoresis. Subsequently, the gels were fixed in 40% (v/v) alcohol and 10% (v/v) acetic acid for 30 min, swollen in 10% (v/v) acetic acid for 20 min, and stained with Coomassie brilliant blue G-250. The gels were rinsed with 10% (v/v) acetic acid until the protein spots were distinct from the background. The gels were then stored in deionized water.

### IMAGE ACQUISITION, DATA ANALYSIS, AND PROTEIN IDENTIFICATION

Gels were scanned using an ImageScanner III (GE Healthcare), and images were analyzed using ImageMaster 2D Platinum v7.0 software (GE Healthcare). Gels of three independent biological replicates per treatment were analyzed. Spots were automatically detected and matched, and mismatched and unmatched spots were artificially modified through manual editing. The spot intensities were normalized according to total intensity of valid spots to reduce the differences in the protein loading and gel staining. Tukey’s test (*P* < 0.05) was applied to test the abundance change of the spots. Only spots with volume ratios over 1.5-fold (*P* < 0.05) were selected for MS identification.

Each marked protein was cut from the gel and cleaved with trypsin. Peptide identification was performed using a 5800 MALDI-TOF/TOF mass spectrometer (AB SCIEX) according to the protocol described by Kosová et al. (2013). The obtained peak list was used to search the databases NCBI *Medicago* (3967), downloaded on June 12, 2014, NCBI Viridiplantae (973373), downloaded on September 13, 2013, and Uniprot (540732), downloaded on September 3, 2013, using MASCOT V2.2 software. Database searches were conducted using the following parameters: peptide mass tolerance ± 100 ppm; fragment mass tolerance ± 0.4 Da; a maximum of one missed cleavage; cysteine carbamidomethylation allowed as a fixed modification; and oxidation of methionine allowed as a dynamical modification. Only significant hits, as defined by the MASCOT probability analysis (*P* < 0.05) with a protein score CI % greater than 95 and a protein score above 50, were accepted. A functional classification of proteins was performed based on the Gene Ontology database^1^ and the Uniprot database^2^.

### STATISTIC ANALYSIS

The data shown in image analyses (holoproteins and low-abundance) and the differential abundance of proteins in the three samples under evaluation (control, 12 h and 7 days) were both compared by analysis of variance (ANOVA) followed by Tukey’s multiple comparison test (*P* < 0.05). Cluster analysis was used to show identified proteins visually changed in the relative abundance using the software PermutMatrix v1.9.3 (Caraux and Pinloche, 2005). The average values of three biological replicates were used to compare the protein changes among different treatments.

## RESULTS

### IMAGE ANALYSES OF HOLOPROTEINS AND LOW-ABUNDANCE PROTEINS OF alfalfa

**FIGURE 1:**
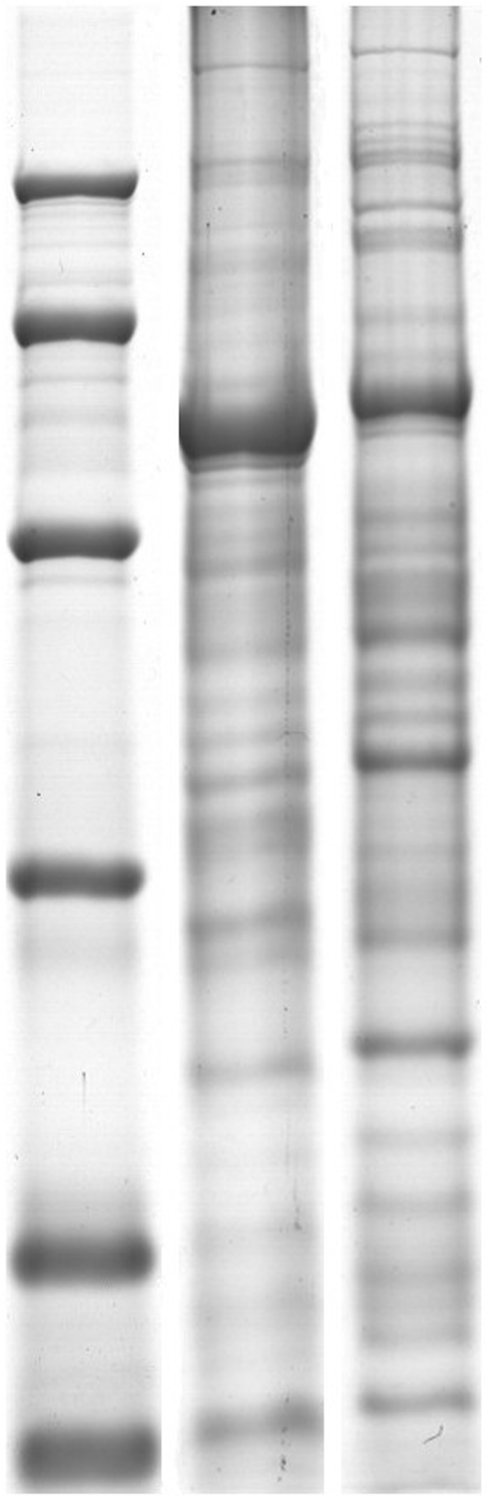
**SDS-PAGE of contrast of holoproteins and low-abundant proteins. (A)** Are holoproteins extracted by TCA/acetone. **(B)** Are low-abundant proteins extracted by PEG. 12.5% SDS-PAGE gel showing RuBisCO depletion. RuBisCO large subunit (LSU) and small subunit (SSU) are marked.

**FIGURE 2:**
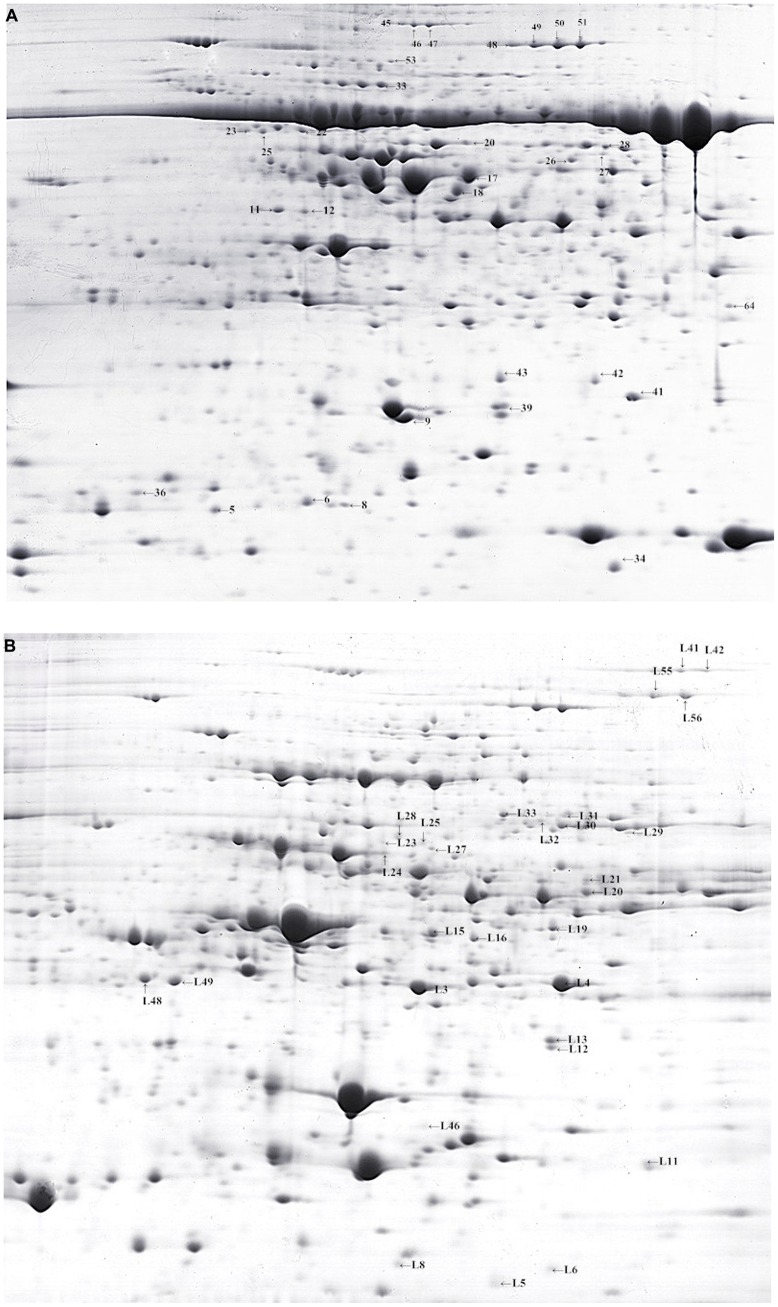
**Two-dimensional gel electrophoresis of *Medicago sativa* leaves (control of W5). (A,B)** 2-DE gels showing identified spots of holoproteins and low-abundant proteins, respectively.

In our study, both holoproteins and low-abundance proteins of alfalfa were extracted to analyze protein changes during chilling stress. The SDS-PAGE result (**Figure 1**) revealed that 17.5% PEG could remove most RuBisCO large subunits; new electrophoretic bands containing low-abundance proteins were clearly detected. Image analysis revealed approximately 498 ZD holoprotein spots and 516 W5 holoprotein spots (**Figure 2A**). Approximately 423 spots were detected in the ZD low-abundance proteome, of which 308 spots were unique to low-abundance versus holoproteins; the ratio of low-abundance protein spots to holoprotein spots was ∼61.85%. In the W5 proteome, this ratio was 64.13%; approximately 320 of 499 spots were unique to low-abundance versus holoproteins (**Figure 2B**). PEG fractionation was useful for the resolution of low-abundance proteins. The detection of new low-abundance proteins aided our analysis of protein variations and adaptive mechanisms during chilling.

### PROTEOMIC DIFFERENCES IN alfalfa IN RESPONSE TO LOW TEMPERATURE

**FIGURE 3:**
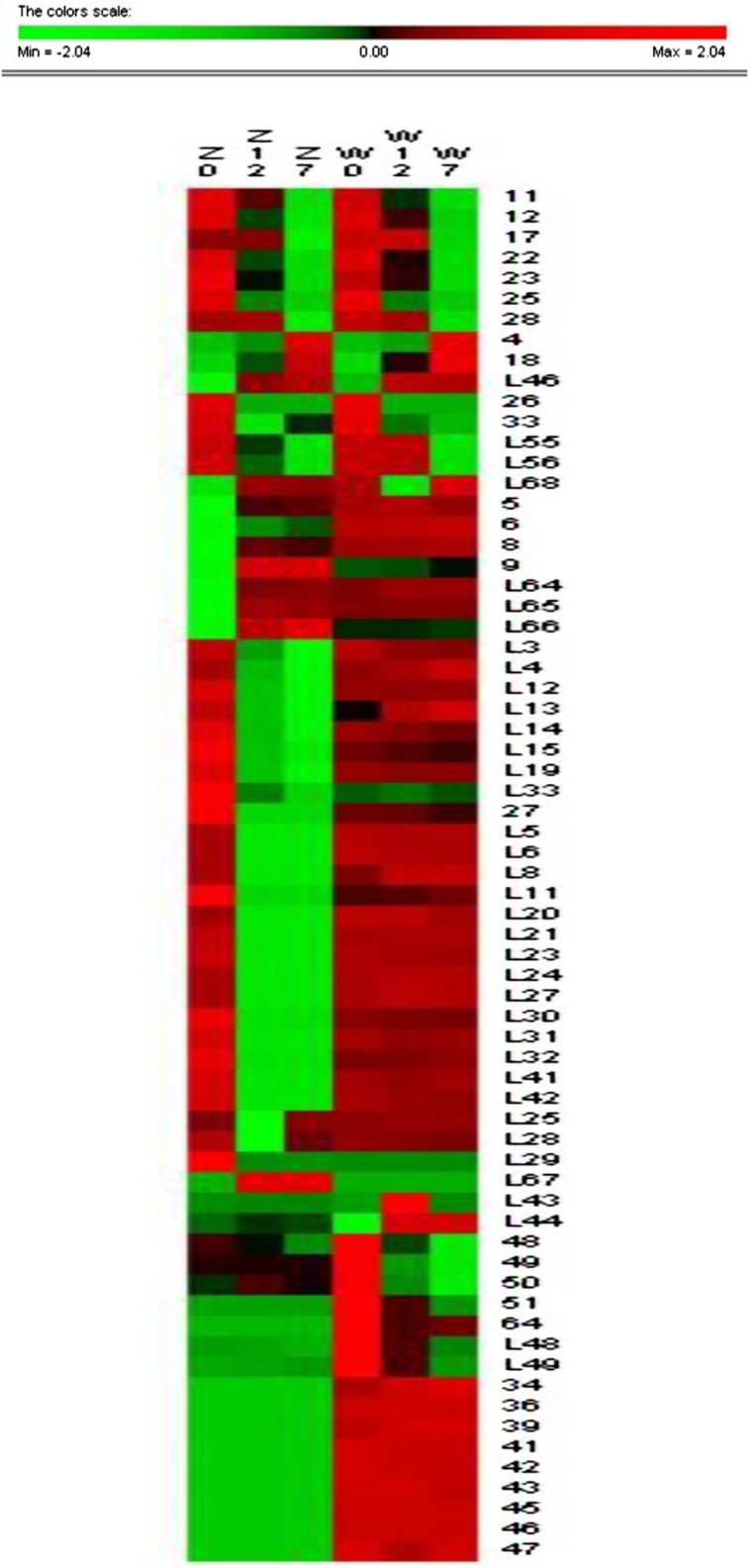
**Cluster analysis of 67 identified spots.** Cluster analysis reveals changes of the relative abundance of identified protein by software PermutMatrix v1.9. Normalized rows (Z scores) and Ward’s algorithm have been used to analyze data. In the figure, ZC is control of ZD, Z12 is treatment of ZD at 4°C for 12 h, Z7 is treatment of ZD at 4°C for 7 days, WC is control of W5, W12 is treatment of W5 at 4°C for 12 h, and W7 is treatment of W5 at 4°C for 7 days. Digits in the figure represent spots No.

To detect differences in cold acclimation between freezing-tolerant alfalfa and freezing-sensitive alfalfa, the variety and relative abundance of proteins were analyzed in two alfalfa cultivars (ZD and W5) in three different growth conditions (control, 4°C for 12 h, and 4°(C for 7days). Holoproteins and low-abundance proteins were extracted from leaves. Two-dimensional gel electrophoresis (2-DE) revealed that a total of 84 spots were differentially expressed at ratios over 1.5-fold in relation to cold acclimation. Of these, 67 spots were successfully identified by MALDI_TOF/TOF (**Table 1**). Clustering analysis (**Figure 3**) was used to visually describe changes of 67 spots in relative abundance during chilling using PermutMatrix software v1.9.3. Compared with W5, more spots were identified in ZD as responding to low temperature: the relative abundance of 49 spots changed in ZD, whereas 24 spots changed in W5. Of these, 15 spots changed in both cultivars in response to low temperature. Compared with controls, chilling (12 h) caused a significant up-accumulation of 13 spots in ZD and six spots in W5, and 34 spots were down-accumulated in ZD (spots 17 and 28 unchanged) and 14 spots in W5 (spots 17, 28, L55 and L56 unchanged). Compared with chilling for 12 h, chilling for 7 days caused an up-accumulation of seven spots in ZD (spots 4, 18, 26, 33, L25, L28, and L46) and five spots (spots 4, 18, 26, L46 and L68), 13 spots (spots 17, 28, L3∼L4, L12∼L15, L19, L29, L33, and L55∼L56) in ZD and eight spots (spots 48∼51, L43 and L48∼L49) in W5 were down-regulated. In additional, nine unchanged spots were found in W5 with a greater relative abundance than in ZD. Chilling affected the relative abundance and variety of proteins. The number of changed proteins were more at 4°C for 12 h than for 7 days.

**Table 1:** The list of 67 indentified spots by MALDI-TOF/TOF analysis.

Spots no	Protein name	Accession no. in NCBI/Uniprot	Plant species	PW (Da)/PI	Pep count	Protein score	Protein score CI %	Intensity matched
**Photosynthesis**
4	RuBisCO small subunit	gi| 16224234	*Medicago sativa*	12078/6.15	8	60	99.612	3.963
5	RuBisCO large subunit	gi| 131991	*M. sativa*	53048.6/6.09	8	88	100	7.728
6	RuBisCO small subunit	gi| 3914601	*M. sativa*	20466.4/8.86	5	292	100	23.873
8	RuBisCO small subunit	gi| 16224234	*M. sativa*	12078/6.15	5	56	99.048	2.943
9	RuBisCO large subunit	gi| 1223773	*M. sativa*	50684.6/6.22	9	234	100	25.61
17	RuBisCO activase, partial	gi| 23320705	*M. sativa*	30170.2/5.63	9	172	100	12.782
18	RuBisCO activase, partial	gi| 23320705	*M. sativa*	30170.2/5.63	10	371	100	30.501
22	Ribulose bisphosphate carboxylase/oxygenase activase B, chloroplastic	RCAB_HORVU	*Hordeum vulgare*	47425.8/7.59	10	434	100	23.233
23	RuBisCO activase, partial	gi| 23320705	*M. sativa*	30170.2/5.63	10	54	98.308	6.412
25	Ribulose bisphosphate carboxylase/oxygenase activase 1, chloroplast precursor, putative	gi| 223536483	*Ricinus communis*	52191.7/5.35	16	275	100	22.017
33	RuBisCO large subunit-binding protein subunit beta, chloroplastic	RUBB_PEA	*Pisum sativum*	63287.4/5.85	16	60	99.555	3.41
34	Ribulose bisphosphate carboxylase small subunit, chloroplastic	gi| 3914601	*M. sativa*	20466.4/8.86	8	248	100	35.996
36	Ribulose 1,5-biphosphate carboxylase large subunit	gi| 1223773	*M. sativa*	50684.6/6.22	3	76	99.989	14.783
39	Ribulose-1,5-bisphosphate carboxylase/oxygenase large subunit, partial	gi| 313664305	*M. sativa*	22508.4/5.76	3	125	100	35.47
41	Ribulose-1,5-bisphosphate carboxylase/oxygenase large subunit, partial	gi| 404243904	*M. sativa*	21709.9/5.78	4	173	100	42.741
42	Chlorophyll A/B binding protein, putative	gi| 223526931	*R. communis*	26644.5/5.96	3	93	100	11.113
43	Predicted: chlorophyll a-b binding protein 6, chloroplastic-like isoform X2	gi| 502177055	*Cicer arietinum*	26490.6/6.96	6	270	100	17.055
64	Ribulose 1,5-biphosphate carboxylase large subunit	gi| 1223773	*M. sativa*	50684.6/ 6.22	24	72	94.268	5.409
L6	Ribulose-1,5-bisphosphate carboxylase small subunit	gi| 16224234	*M. sativa*	12078/6.15	9	505	100	44.248
L8	Cytochrome b6-f complex iron-sulfur subunit chloroplastic	UCRIA_PEA	*P. sativum*	24683.4/8.63	3	183	100	6.296
L23	RuBisCO activase, partial	gi| 23320705	*M. sativa*	30170.2/5.63	17	391	100	23.23
L43	Ribulose-1,5-bisphosphate carboxylase/oxygenase large subunit, partial	gi| 404243904	*M. sativa*	21709.9/5.78	3	172	100	12.011
L64	Oxygen-evolving enhancer protein 1, chloroplastic	gi| 131384	*P. sativum*	35099.9/6.25	10	359	100	25.226
L65	Oxygen-evolving enhancer protein 1, chloroplastic	gi| 131384	*C. arietinum*	35099.9 /6.25	10	358	100	20.313
L67	Oxygen-evolving enhancer protein 1, chloroplastic-like	gi| 502153108	*C. arietinum*	35106.8 /6.24	8	281	100	18.204
L68	Predicted: oxygen-evolving enhancer protein 1, chloroplastic-like	gi| 502153108	*C. arietinum*	35106.8/ 6.24	8	378	100	29.231
**Energy metabolism**
48	Predicted: transketolase, chloroplastic-like	gi| 502121526	*C. arietinum*	80412.5/6	8	184	100	16.349
49	Predicted: transketolase, chloroplastic-like	gi| 502121526	*C. arietinum*	80412.5/ 6	17	274	100	21.246
50	Predicted: transketolase, chloroplastic-like	gi| 502121526	*C. arietinum*	79905.7/ 6.51	6	275	100	20.772
51	Predicted: transketolase, chloroplastic-like	gi| 502121526	*C. arietinum*	80412.5 /6	17	257	100	22.668
53	Predicted: V-type proton ATPase catalytic subunit A-like	gi| 502149512	*C. arietinum*	68929.9/5.25		753	100	40.526
L3	Predicted: triosephosphate isomerase, chloroplastic-like	gi| 502111535	*C. arietinum*	33799.4/6.36	13	528	100	29.197
L4	Rriosephosphate isomerase	gi| 351721638	*Glycine max*	27441.3/5.87	4	158	100	6.294
L20	Malate dehydrogenase precursor	gi| 2827080	*M. sativa*	36003.1/8.8	9	152	100	4.615
L24	Predicted: phosphoribulokinase, chloroplastic-like	gi| 502162280	*C. arietinum*	45815.3/6.41	17	331	100	27.518
L25	Predicted: adenosine kinase 2-like	gi| 502121977	*C. arietinum*	37997.9/5.29	5	87	99.819	3.813
L27	Predicted: sedoheptulose-1,7-bisphosphatase,chloroplastic-like	gi| 502137914	*C. arietinum*	42165.5/ 6.35	14	81	99.997	3.225
L41	Predicted: aconitate hydratase 2, mitochondrial-like	gi| 502083283	*C. arietinum*	108010.7/7.59	26	508	100	31.555
L42	Predicted: aconitate hydratase 2, mitochondrial-like	gi| 502183208	*C. arietinum*	108255.2/7.89	25	550	100	36.642
**Stress and redox**
27	Monodehydroascorbate reductase	gi| 369726464	*M. sativa*	47243.5/6.3	16	260	100	13.456
L11	Glutathione peroxidase, partial	gi| 401716808	*M. sativa*	24886/9.24	9	304	100	13.66
L12	Peptide methionine sulfoxide reductase A3	MSRA3_ARATH	*Arabidopsis thaliana*	22815.8/5.34	5	283	100	40.34
L13	Peptide methionine sulfoxide reductase A3	MSRA3_ARATH	*A. thaliana*	22825.8/5.64	7	289	100	40.56
L14	Thioredoxin-like protein CDSP32, chloroplastic	CDSP_ARATH	*A. thaliana*	33948.5/8.65	7	139	100	20.946
L15	Thioredoxin-like protein CDSP32, chloroplastic	CDSP_ARATH	*A. thaliana*	33948.5/8.65	8	158	100	26.501
L21	Predicted: probable aldo-keto reductase 2-like	gi| 502102350	*C. arietinum*	38423.4/5.817	7	200	100	5.647
L32	Monodehydroascorbate reductase	gi| 369726464	*M. sativa*	47243.5/6.3	19	344	100	18.945
L48	Predicted: 2-Cys peroxiredoxin BAS1-like, chloroplastic-like isoform X1	gi| 502112098	*C. arietinum*	30698.1/6.12	10	282	100	29.729
L49	Predicted: 2-Cys peroxiredoxin BAS1-like, chloroplastic-like isoform X2	gi| 502112102	*C. arietinum*	29144.1/6.12	10	281	100	45.543
L66	Predicted: 2-Cys peroxiredoxin BAS1-like, chloroplastic-like isoform X2	gi| 502112102	*C. arietinum*	29144.1/6.12	7	277	100	36.298
**Protein folding and disassembling**
45	Predicted: chaperone protein ClpC, chloroplastic-like isoform X1	gi| 502139520	*C. arietinum*	102754.1/6.37	29	331	100	35.226
46	Predicted: chaperone protein ClpC, chloroplastic-like isoform X2	gi| 502133388	*C. arietinum*	102968.1/6.35	28	541	100	37.689
47	Chaperone protein ClpC Precursor, chloroplastic	gi| 461753	*P. sativum*	102818.1/6.55	29	316	100	29.553
L5	GTPase obg	OBG_PSYCK	*Psychrobacter cryohalolentis*	44308.6/4.86	15	71	95.705	4.081
L44	peptidyl-prolyl *cis*-*trans* isomerase CYP20-3	gi| 334186198	*A. thaliana*	28403.2/8.97	3	88	99.831	3.552
L46	Eukaryotic translation initiation factor 5A-2	gi| 20138664	*M. sativa*	17501.7/5.41	7	135	100	8.896
**Biosynthesis**
26	Glutamate 1-semialdehyde aminotransferase	gi| 345451030	*M. sativa*	50260.5/6.36	15	466	100	39.627
L19	Predicted: cinnamoyl-CoA reductase 1-like	gi| 502129455	*C. arietinum*	35160/5.49	10	358	100	22.308
L29	Glutamate 1-semialdehyde aminotransferase	gi| 345451030	*M. sativa*	50260.5/6.36	17	552	100	55.513
L30	Glutamate 1-semialdehyde aminotransferase	gi| 345451030	*M. sativa*	50260.5/6.36	11	191	100	10.027
L31	Predicted: 1-deoxy-D-xylulose 5-phosphate reductoisomerase, chloroplastic-like	gi| 502155936	*C. arietinum*	52651.6/6.24	11	125	100	9.375
**Amino acid metabolism**
28	*S*-adenosyl-L-methionine synthetase	gi| 139478060	*M. falcata*	43588/5.77	9	100	100	8.508
L33	*S*-adenosyl-L-methionine synthetase	gi| 139478060	*M. falcata*	43588/5.77	12	301	100	34.57
L55	5-methyltetrahydropteroyltriglutamate-homocysteinemethyltransferase	METE_ARATH	*A. thaliana*	84645.6/6.09	11	460	100	20.829
L56	5-methyltetrahydropteroyltriglutamate-homocysteinemethyltransferase-like	gi| 525345100	*C. arietinum*	84599.7/6.01	20	713	100	26.846
**Others**
11	Predicted: uncharacterized protein LOC101499502	gi| 502140419	*C. arietinum*	36689.6/6.3	11	222	100	24.257
12	Unnamed protein product	gi| 297746499	*Vitis vinifera*	24323.4/7.63	5	68	95.924	4.256
L28	Unnamed protein product	gi| 257737972	*M. sativa*	38746.3/5.76	8	322	100	9.297

**FIGURE 4:**
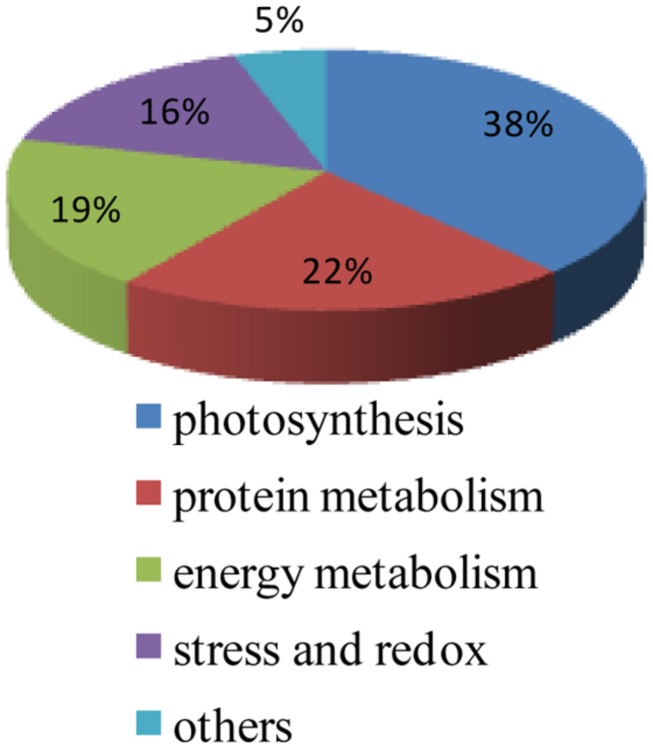
**Functional classification of 67 protein spots responding to cold acclimation.** The 67 indentified spots include proteins involved in photosynthesis (26 spots), protein metabolism (15 spots), energy metabolism (13 spots), stress and redox (10 spots), and other functions (three spots).

Protein functional analysis has been carried out according to Gene Ontology database and the Uniprot database. Based on their functional features, the 67 differentially expressed proteins were classified into five categories, as follows: photosynthesis, protein metabolism, energy metabolism, stress and redox, and other functions (**Figure 4** and **Table 1**). Among these protein spots, 11 changed in a similar manner in both ZD and W5 under cold stress. Compared with control, the relative abundance of uncharacterized protein LOC101499502 (spot 11), unnamed protein product (spot 12), and RuBisCO activase (spots 22, 23, and 25) declined at 12 h and that of RuBisCO activase (spots 17 and 28) declined at 7 days, the relative abundance of RuBisCO small subunit (spot 4), RuBisCO activase (spot 18) and eukaryotic translation initiation factor 5A-2 (eIF-5A, spot L46) continuously increased at 12 h and 7 days, and glutamate 1-semialdehyde aminotransferase (spot 26) was down-regulated at 12 h and up-regulated at 7 days. The expression patterns of RuBisCO large subunit-binding protein subunit beta (spot 33), 5-methyltetrahydropteroyltriglutamate-homocysteinemethyltransferase (spots L55 and L56), and oxygen-evolving enhancer protein (spot L68) were altered in different ways in ZD and W5 (**Figure 3**). During chilling, the relative abundance of the remaining 32 spots changed in ZD and remained constant in W5. Of these, RuBisCO large subunit (spots 5 and 9), RuBisCO small subunit (spots 6 and 8) and oxygen-evolving enhancer protein (spots L64∼L66) were up-regulated at 12 h, and triosephosphate isomerase (spots L3 and L4), peptide methionine sulfoxide reductase (spots L12 and L13), thioredoxin-like protein (spots L14 and L15), cinnamoyl-CoA reductase (spot L19) and *S*-adenosyl-L-methionine synthetase (spot L33) were continuously down-regulated at 12 h and 7 days. Monodehydroascorbate reductase (spots 27 and L32), GTPase obg (spot L5), RuBisCO small subunit (spot L6), cytochrome b6-f complex iron-sulfur subunit (spot L8), glutathione peroxidase (spot L11), malate dehydrogenase precursor (spot L20), aldo-keto reductase (spot L21), RuBisCO activase (spot L23), phosphoribulokinase (spot L24), sedoheptulose-1, 7-bisphosphatase (spot L27), glutamate 1-semialdehyde aminotransferase (spot L30), 1-deoxy-D-xylulose 5-phosphate reductoisomerase (spot L31) and aconitate hydratase 2 (spots L41 and L42) maintained the same lower relative abundance at 12 h and 7 days compared with the control. The relative abundance of adenosine kinase 2 (spot L25) and unnamed protein product (spot L28) decreased at 12 h and increased at 7 days. Two spots were expressed exclusively in ZD: the relative abundance of glutamate 1-semialdehyde aminotransferase (spot L29) decreased and that of oxygen-evolving enhancer protein (spot L67) increased. In W5, only nine proteins spots were differentially expressed and remained unchanged in ZD. RuBisCO large subunit (spot L43) and peptidyl-prolyl *cis*-*trans* isomerase (PPlase, spot L44) were up-regulated, transketolase (spots 48∼51) and 2-Cys peroxiredoxin (spots L48 and 49) were down-regulated at 12 h and 7 days, and RuBisCO large subunit (spot 64) was only down-regulated at 12 h. In additionally, RuBisCO small and large subunit (spots 34, 36, 39, and 41), chlorophyll A/B binding protein (spots 42 and 43) and chaperone protein ClpC (spots 45∼47) were only highly expressed in W5 under chilling stress.

Cold acclimation in plant is a complex progress and involved in various proteins. In our study, photosynthesis related proteins were the largest category of differentially expressed in both ZD and W5. A series of changes between RuBisCO activase (spots 17, 18, 22, 23, 25, and L23) and RuBisCO large and small subunits (spots 4, 5, 6, 8, 9, 64, L6, and L43) were found in ZD and W5 in response to low temperature. Compared to freezing-sensitive alfalfa, a greater number of proteins were changed in freezing-tolerant alfalfa under chilling stress. In ZD, protein metabolism, energy metabolism and stress and redox related proteins were influenced, such as *S*-adenosyl-L-methionine synthetase (spot L33), GTPase obg (spot L5), glutathione peroxidase (spot L11), malate dehydrogenase precursor (spot L20), and so on. Autologous metabolism and biosynthesis were slowed to response low temperature in ZD. Increased PPIase and eIF-5A is consistent with an increased capability for protein folding and protein biosynthesis, suggesting increased protection against chilling stress in W5.

## DISCUSSION

### THE REMOVAL OF “OVERABUNDANT” PROTEINS

RuBisCO exists in most green plants and comprises 30∼60% of the total protein. RuBisCO is an important photosynthesis enzyme that fixes CO_2_ in the Calvin cycle (Ellis, 1979; Voet and Voet, 1995; Whitney and Andrews, 2001; Parry et al., 2003; Giavalisco et al., 2005). As an “overabundant” protein, RuBisCO influences the detection of some low-abundance proteins. PEG, at a proper concentration, effectively removes most large subunits of RuBisCO and improves the dynamic resolution of low-abundance proteins in different plant species. In *Arabidopsis thaliana*, 16% PEG was applied to extract low-abundance proteins using a fractionation method; 80% more spots were revealed compared to a holoprotein sample extracted using a TCA/acetone method (Xi et al., 2006). Lee et al. (2007) suggested that 15% PEG could be used for the effective extraction of low-abundance protein to analyze protein changes in rice leaves under cold stress. In our study, 17.5% PEG significantly reduced the masking effect of RuBisCO, and new proteins were detected in both ZD and W5. Thirty-five out of 67 spots were low-abundance proteins in response to chilling stress (**Table 1**). These data could help us to better understand cold adaptation mechanisms in alfalfa.

### PROTEINS CHANGES IN PROTEOME

#### Differentially expressed photosynthetic proteins

Proteomic analysis revealed that different cold acclimation mechanisms exist in ZD and W5. In some cases, the same protein exhibited differential accumulation patterns in alfalfa cultivars with different tolerance to cold stress. In our study, the largest category of proteins that responded to chilling in both ZD and W5 is photosynthesis-related proteins. It has been reported the interaction between RuBisCO activase and RuBisCO large and small subunits (Portis, 1990; Spreitzer and Salvucci, 2002). When *RdreB1BI* transgenic strawberries are exposed to low temperature, RuBisCO activase reactivates RuBisCO to fix any remaining CO_2_ and protect the dissipation of energy from the photo respiratory oxygenase reaction (Haupt-Herting et al., 2001; Gua et al., 2013). In alfalfa, RuBisCO activase (spot 18) increased in both ZD and W5; the expression of this protein was higher in ZD than that in W5 at 4°C for 7 days. A greater number of up-regulation of spots corresponding to the large and small subunits of RuBisCO (spots 4, 5, 6, 8, and 9) were found in ZD at 12 h, compared to W5 (spots 4 and L43). The abundance of the RuBisCO large subunit-binding protein subunit β (spot 33) decreased for the correct assembly of the RuBisCO holoenzyme in W5; a similar result was observed in pea (Barraclough and Ellis, 1980; Grimaud et al., 2013). Chilling stress also affected the relative abundance of RuBisCO activase (spots 17, 22, 23, 25, and L23). The relative abundance of the RuBisCO small subunit (spot L6) decreased in ZD, and RuBisCO large subunit (spot 64) decreased in W5. The disassembly of RuBisCO may reduce the photosynthetic rate under cold stress (Yan et al., 2006; An et al., 2011; Zhang et al., 2012). The series of changes between RuBisCO activase and RuBisCO large and small subunits could make the active site more accessible for carbamylation, thereby enhancing CO_2_ fixation to resist cold stress (Portis et al., 2008). The relative abundance of the cytochrome b6-f complex iron-sulfur subunit (spot L8) declined in ZD as in *Thellungiella* rosette and in a cold-tolerant Champagne cultivar of pea, which affected electron transport from PSII to PSI during cold acclimation (Gao et al., 2009; Grimaud et al., 2013); whereas its expression remained unchanged in W5. Oxygen-evolving enhancer protein 1 (spots L64, L65, L67, and L68) increased in ZD, which stabilizes the tetranuclear manganese (Mn) center that is the location of photoinhibition in PSII (Sarvikas et al., 2006). In addition to L68, abundance of the other three oxygen-tetranuclear Mn center proteins (L64, L65, and L67) was unchanged in W5. As in a freeze-tolerant genotype of *Festuca pratensis*, oxygen-evolving enhancer protein 1 (L68) in W5 was degraded at 12 h and accumulated at 7 days. Kosmala et al. (2009) postulated that this change may contribute to additional metabolic disturbances rather than lower susceptibility to photoinhibition.

Compared to W5, more proteins were mobilized in ZD during adaptation to low-temperature stress. The relative abundance of photosynthesis-related proteins in ZD was altered in a complex manner to relieve the influence of chilling stress on photosynthesis. However, the abundance of these proteins remained relatively stable in W5. The RuBisCO small subunit (spot 34) and large subunit (spots 36, 39, and 41) were only highly expressed in W5. The greater relative abundance of chlorophyll A/B binding protein (spots 42 and 43), a component of the light-harvesting complex, allows for the absorption of more light by chlorophyll excitation, and the transfer of energy to the photochemical reaction centers allows for adaptation to chilling stress in W5 (Green and Durnford, 1996).

#### Differentially expressed metabolism proteins

The category of protein metabolism involved proteins related folding and disassembling, biosynthesis and amino acid metabolism. Chilling induced eIF-5A (spot L 46) accumulation in both ZD and W5 as in wheat and *Thellungiella* rosette (Gao et al., 2009; Vítamìvasì et al., 2012; Kosová et al., 2013). EIF-5A is mainly involved in RNA metabolism, protein translation and the regulation of the cell cycle (Schatz et al., 1998; Thompson et al., 2004; Jao and Chen, 2006; Feng et al., 2007). Kosová et al. (2013) indicated that these changes were associated with protein expression and protein regulation and reflect more profound changes at the regulatory level in cold-treated plants. Cold affected the *S*-adenosylmethionine (SAM) synthetic pathway in both ZD and W5. In this pathway, methionine production is catalyzed by methionine synthase with assistance of the coenzyme 5-methyltetrahydropteroyltriglutamate-homocysteine methyltransferase (spots L55 and L56). *S*-adenosyl-L-methionine synthetase (spots 28 and L33) then transfers adenosine to catalyze SAM. Proteomic studies have demonstrated that SAM plays an important role in cold stress resistance (Cui et al., 2005; Kosová et al., 2013). The observed reduction in the relative abundance of spots L55 and L56 began at 12 h in ZD and at 7 days in W5. These findings suggest that cold stress affects the activity of 5-methyltetrahydropteroyltriglutamate-homocysteine methyltransferase, as reported in rice seedlings (Hashimoto and Komatsu, 2007). *S*-adenosyl-L-methionine synthetase was consumed to produce SAM, preventing cold injury in alfalfa, although the reduction in the coenzyme led to an insufficient supply of substrate. Chilling had a clear influence on amino acid metabolism in alfalfa, leading to a reduction in amino acid synthesis for energy conservation during low temperature adaptation.

GTPase obg (spot L5) is a conserved GTPase binding protein mainly involved in cell proliferation, cell development, signal transduction, and protein translation (Bourne et al., 1990; Kaziro et al., 1991). Through an analysis of *obgc* mutants in *Arabidopsis* and rice, Bang et al. (2012) found that plastid rRNA processing is defective, indicating that *obgc* functions primarily in plastid ribosome biogenesis during chloroplast development. In ZD, it was speculated that the decreased relative abundance of GTPase obg during chilling might impede the synthesis of a series of related proteins. Biosynthesis-related enzymes were also significantly influenced by cold stress. Glutamate 1-semialdehyde aminotransferase (spots 26, L29, and L30) catalyzes the conversion of glutamate-1-semialdehyde to aminolevulinic acid, which is a step in the assembly of chlorophyll, coenzyme B12, heme, and other tetrapyrrolic proteins (Jordan and Shemin, 1972; Gough et al., 2001). Cinnamoyl-CoA reductase (CCR, spot L19) catalyzes the first step in monolignol biosynthesis and plays a key role in synthesis of lignin (Zhou et al., 2010). The protein 1-deoxy-D-xylulose 5-phosphate reductoisomerase (spot L31) is a key rate-limiting enzyme and the regulatory site of terpenoid synthesis (Takahashi et al., 1998). The relative abundance of these spots decreased in ZD, and with the exception of spot 26, the expression of these spots remained unchanged in W5. Chilling stress was clearly more severe in ZD than in W5, as three biosynthetic pathways were disrupted in ZD.

In W5, the expression of PPlase (spot L44) increased under cold stress. This is similar to a finding in *Populus cathayana* males (Zhang et al., 2012), indicating that these proteins are involved in protein folding to overcome stress (Budiman et al., 2011). Additionally, high expression of the chaperone protein ClpC (spots 45, 46, and 47) was only observed in W5, which regulated protein metabolism and kept homeostasis. As a protein chaperone, ClpC is mainly involved in regulating the structure and function of many polypeptides and hydrolyzes irreversibly damaged proteins to prevent the accumulation of potentially cytotoxic polypeptides (Parsell and Lindquist, 1993).

#### Differentially expressed energy metabolism proteins

The third functional category of proteins that respond to low temperatures in alfalfa involve energy metabolism such as the Calvin cycle, Kreb’s cycle, and glycolysis pathways. Chilling had a large effect on carbon metabolism (phosphoribulokinase, spot L24, and sedoheptulose-1, 7-bisphosphatase, spot L27), ATP production (malate dehydrogenase precursor, spot L20, and aconitate hydratase 2, spots L41 and L42) and glycolysis (triosephosphate isomerase, spots L3 and L4) in ZD. Transketolase (spots 48∼51), which is involved in the Calvin cycle were down-regulated in W5. Unlike cold-tolerant pea, transketolase was more highly expressed in W5 compared to ZD, whereas this protein was constitutively expressed in ZD under cold stress (Grimaud et al., 2013). In addition, a reduction in the abundance of adenosine kinase 2 (spot L25) was shown to disturb the regulation of energy metabolism and the balance of ATP, ADT, and AMP at the beginning of chilling in ZD (Veuthey and Stucki, 1987; Zeleznikar et al., 1995). The increase in adenosine kinase 2 expression allowed for recovered activity and the maintenance of the energy balance in the cell at 7 days. We deduced that a decrease in the abundance of several enzymes leads to slowed energy metabolism as energy is stored for maintaining body balance in alfalfa.

#### Differentially expressed stress and redox proteins

In plants, cellular redox homeostasis can be disturbed by cold stress. Such disturbances result in the production of ROS, which stimulate oxidative damage in the organism. Therefore, certain ROS-scavenging enzymes play an important role in the scavenging of ROS and maintaining iron balance. Glutathione peroxidase (spot L11; Liu et al., 2014), thioredoxin-like protein (spots L14 and L15; Zhang et al., 2012; Kosová et al., 2013), probable aldo-keto reductase 2 (spot L21; Gao et al., 2009; Koehler et al., 2012) and 2-Cys peroxiredoxin (spots L48, L49, and L66; Yan et al., 2006; Gao et al., 2009) were up-regulated under cold stress. Their functions are mainly involved in detoxication, protection from oxidative damage, preventing the membrane lipid from peroxidation and enhancing tolerance to ROS (Bartels, 2001; Kim et al., 2011). Monodehydroascorbate reductase (spot 27 and L32) is an important enzyme involved in the regeneration of ascorbic acid for scavenging hydrogen peroxide (Arrigoni et al., 1981), and peptide methionine sulfoxide reductase A3 (spots L12 and L13) may repair oxidatively damaged proteins *in vivo* (Brot et al., 1982a,b). To resist cold stress, ZD consumes abundant ROS-scavenging enzymes to maintain cellular redox homeostasis and protect the body. The relative abundance of 2-Cys peroxiredoxin (spot L66) increased; this protein catalyzes the reduction of various hydroperoxide to the corresponding alcohol or water and detoxifies alkyl hydroperoxides and peroxinitrite (Dietz et al., 2006; Kim et al., 2011). The relative abundance of 2-Cys peroxiredoxin (spots L48 and L49) declined in W5, whereas other ROS-scavenging enzymes were not significantly affected by low temperature. It may be inferred that ZD is more sensitive to chilling stress than W5 in spite of its freeze-tolerant genotype. When chilling (4°C) occurs over a short period (such as 12 h), ZD consumes enzymes to resist stress. The enzyme expression profile then changed to maintain homeostasis, as indicated by a low relative abundance observed at 7 days. The observed response to chilling in ZD was more drastic and complex than in W5.

As a freezing-tolerant genotype, ZD has stronger cold tolerance than the freezing-sensitive cultivar W5. However, ZD is more sensitive to chilling than W5, and a greater number of changes were observed in proteins involved in photosynthesis, energy metabolism, stress and redox, biosynthesis metabolism and amino acid metabolism in ZD under chilling stress. On one hand, more enzymes were consumed to produce proteins for regulating metabolism and maintaining homeostasis in ZD. On the other hand, low temperature influenced enzymatic activity and altered the metabolic and synthesis pathways. In W5, the expression of many proteins remained unchanged. However, protein metabolism-related proteins were more active in W5 compared to ZD. In conclusion, ZD mobilizes a large number of proteins to adapt low temperature, and autologous metabolism and biosynthesis are slowed to reduce consumption for homeostasis. W5 enhances its capability for protein folding and protein biosynthesis to overcome chilling stress. The perception of low temperature is more sensitive in freezing-tolerant alfalfa than in freezing-sensitive alfalfa. Proteomics provides new insight into cold acclimation mechanisms in alfalfa.

## Conflict of Interest Statement

The authors declare that the research was conducted in the absence of any commercial or financial relationships that could be construed as a potential conflict of interest.

## Footnotes

1 http://www.geneontology.org

2 http://www.uniprot.org

## References

[B1] Amme S. Matros A. Schlesier B. Mock H. P. (2006). Proteome analysis of cold stress response in *Arabidopsis thaliana* using DIGE-technology. *J. Exp. Bot.* 57 1537–1546 10.1093/jxb/erj129 16574749

[B2] An B. Y. Liu X. Y. Tan H. Lin W. H. Sun L. W. (2011). Comparative profile of RuBisCO-interacting proteins from *Arabidopsis*: photosynthesis under cold conditions. *Prog. Biochem. Biophys.* 38 455–463 10.3724/SP.J.1206.2011.000009

[B3] Arrigoni O. Dipierro S. Borraccino G. (1981). Ascorbate free radical reductase, a key enzyme of the ascorbic acid system. *FEBS Lett.* 125 242–245 10.1016/0014-5793(81)80729-6

[B4] Balbuena T. S. Salas J. J. Martínez-Force E. Garcés R. Thelen J. J. (2011). Proteome analysis of cold acclimation in sunflower. *J. Proteome Res.* 10 2330–2346 10.1021/pr101137q 21341810

[B5] Bang W. Y. Chen J. Jeong I. S. Kim S. W. Kim C. W. Jung H. S. (2012). Functional characterization of ObgC in ribosome biogenesis during chloroplast development. *Plant J.* 71 122–134 10.1111/j.1365-313X.2012.04976.x 22380942

[B6] Barraclough R. Ellis R. J. (1980). Protein synthesis in chloroplasts IX. Assembly of newly-synthesized large subunits into ribulose bishopshate carboxylase in isolated intact pea chloroplasts. *Biochim. Biophys. Acta* 608 19–31 10.1016/0005-2787(80)90129-X 7388030

[B7] Bartels D. (2001). Targeting detoxification pathways: an efficient approach to obtain plants with multiple stress tolerance. *Trends Plant Sci.* 6 284–286 10.1016/S1360-1385(01)01983-5 11435150

[B8] Bocian A. Kosmala A. Rapacz M. Jurczyk B. Marczak Ł. Zwierzykowski Z. (2011). Differences in leaf proteome response to cold acclimation between *Lolium perenne* plants with distinct levels of frost tolerance. *J. Plant Physiol.* 168 1271–1279 10.1016/j.jplph.2011.01.029 21489653

[B9] Bourne H. R. Sanders D. A. McCormick F. (1990). The GTPase superfamily: a conserved switch for diverse cell functions. *Nature* 348 125–132 10.1038/348125a0 2122258

[B10] Brot N. Weissbach L. Werth J. Weissbach H. (1982a). The biochemistry of methionine sulfoxide residues in proteins. *Biofactor* 3 91–96 10.1016/0968-0004(82)90204-3 1910456

[B11] Brot N. Weissbach L. Werth J. Weissbach H. (1982b). Reduction of N-acetyl methionine sulfoxide: a simple assay for peptide methionine sulfoxide reductase. *Anal. Biochem.* 122 291–294 10.1016/0003-2697(82)90283-4 7114447

[B12] Budiman C. Koga Y. Takano K. Kanaya S. (2011). FK506-binding protein 22 from a psychrophilic bacterium, a cold shock-inducible peptidyl prolyl isomerase with the ability to assist in protein folding. *Int. J. Mol. Sci.* 12 5261–5284 10.3390/ijms12085261 21954357 PMC3179164

[B13] Caraux G. Pinloche S. (2005). PermutMatrix: a graphical environment to arrange gene expression profiles in optimal linear order. *Bioinformatics* 21 1280–1281 10.1093/bioinformatics/bti141 15546938

[B14] Carrol A. W. Joshi H. J. Heazlewood J. L. (2013). Managing the green proteomes for the next decade of plant research. *Front. Plant Sci.* 4:501 10.3389/fpls.2013.00501 PMC3864100 24379820

[B15] Castonguay Y. Laberge S. Brummer C. E. Volenec J. J. (2006). Alfalfa winter hardiness: a research retrospective and integrated perspective. *Adv. Agron.* 90 203–265 10.1016/S0065-2113(06)90006-6

[B16] Corthalis G. L. Wasinger V. C. Hochstrasser D. F. Sanchez J. C. (2000). The dynamic range of protein expression: a challenge for proteomic research. *Electrophoresis* 21 1104–1115 10.1002/(SICI)1522-2683(20000401)21:6<1104::AID-ELPS1104>3.3.CO;2-3 10786884

[B17] Cui S. X. Huang F. Wang J. Ma X. Cheng Y. S. Liu J. Y. (2005). A proteomic analysis of cold stress responses in rice seedlings. *Proteomics* 5 3162–3172 10.1002/pmic.200401148 16078185

[B18] Degand H. Faber A. M. Dauchot N. Mingeot D. Watillon B. Cutsemet P. V. (2009). Proteomic analysis of chicory root identifies proteins typically involved in cold acclimation. *Proteomics* 9 2903–2907 10.1002/pmic.200800744 19405027

[B19] Dietz K. J. Jacob S. Oelze M. L. Laxa M. Tognetti V. De Miranda S. M. N. (2006). The function of peroxiredoxins in plant organelle redox metabolism. *J. Exp. Bot.* 57 1697–1709 10.1093/jxb/erj160 16606633

[B20] Dumont E. Bahrman N. R. Goulas E. Valot B. Sellier H. Hilbert J. L. (2011). A proteomic approach to decipher chilling response from cold acclimation in pea (*Pisum sativum* L.). *Plant Sci.* 180 86–98 10.1016/j.plantsci.2010.09.006 21421351

[B21] Ellis R. J. (1979). The most abundant protein in the world. *Trends Biochem. Sci.* 4 241–244 10.1016/0968-0004(79)90212-3

[B22] Feng H. Chen Q. Feng J. Zhang J. Yang X. Zuo J. (2007). Functional characterization of the *Arabidopsis* eukaryotic translation initiation factor 5A-2 that plays a crucial role in plant growth and development by regulating cell division, cell growth, and cell death. *Plant Physiol.* 144 1531–1545 10.1104/pp.107.098079 17513484 PMC1914145

[B23] Gao F. Zhou Y. J. Zhu W. P. Li X. F. Fan L. M. Zhang G. F. (2009). Proteomic analysis of cold stress-responsive proteins in *Thellungiella* rosette leaves. *Planta* 230 1033–1046 10.1007/s00425-009-1003-6 19705148

[B24] Giavalisco P. Nordhoff E. Kreitler T. Kloppel K. D. Lehrach H. Klose J. (2005). Proteome analysis of *Arabidopsis thaliana* by two-dimensional gel electrophoresis and matrix-assisted laser desorption/ionisation-time of flight mass spectrometry. *Proteomics* 5 1902–1913 10.1002/pmic.200401062 15815986

[B25] Gomat H. Y. Deleporte P. Moukini R. Mialounguila G. Ognouabi N. Saya A. R. (2011). What factors influence the stem taper of eucalyptus: growth, environmental conditions, or genetics? *Ann. For. Sci.* 68 109–120 10.1007/s13595-011-0012-3

[B26] Gough K. C. Hawes W. S. Kilpatrick J. Whitelam G. C. (2001). Cyanobacterial GR6 glutamate-1-semialdehyde aminotransferase: a novel enzymebased selectable marker for plant transformation. *Plant Cell Rep.* 20 296–300 10.1007/s002990100337

[B27] Green B. R. Durnford D. G. (1996). The chlorophyll-carotenoid proteins of oxygenic photosynthesis. *Annu. Rev. Plant Physiol. Plant Mol. Biol.* 47 685–714 10.1146/annurev.arplant.47.1.685 15012305

[B28] Grimaud F. Renaut J. Dumont E. Sergeant K. Lucau-Danila A. Blervacq A. S. (2013). Exploring chloroplastic changes related to chilling and freezing tolerance during cold acclimation of pea (*Pisum sativum* L.). *J. Proteomics* 80 145–159 10.1016/j.jprot.2012.12.030 23318888

[B29] Gua X. B. Gao Z. H. Zhuang W. B. Qiao Y. S. Wang X. Y. Mi L. (2013). Comparative proteomic analysis of rd29A: RdreB1BI transgenic and non-transgenic strawberries exposed to low temperature. *J. Plant Physiol.* 170 696–706 10.1016/j.jplph.2012.12.012 23394786

[B30] Hashimoto M. Komatsu S. (2007). Proteomic analysis of rice seedlings during cold stress. *Proteomics* 7 1293–1302 10.1002/pmic.200600921 17380535

[B31] Haupt-Herting S. Klug K. Fock H. P. (2001). A new approach to measure gross CO2 fluxes in leaves. Gross CO2 assimilation, photorespiration, and mitochondrial respiration in the light in tomato under drought stress. *Plant Physiol.* 126 388–396 10.1104/pp.126.1.388 11351101 PMC102312

[B32] Heazlewood J. L. (2011). The green proteome: challenges in plant proteomics. *Front. Plant Sci.* 2:6 10.3389/fpls.2011.00006 PMC3355608 22639573

[B33] Jao D. L. E. Chen K. Y. (2006). Tandem affinity purification revealed the hypusine-dependent binding of eukaryotic initiation factor 5A to the translating 80S ribosomal complex. *J. Cell. Biochem.* 97 583–598 10.1002/jcb.20658 16215987

[B34] Jordan P. M. Shemin D. (1972). *Aminolevulinic Acid Aynthetase, in the Enzymes*. New York: Academic Press.

[B35] Kaur N. Gupta A. K. (2005). Signal transduction pathways under abiotic stresses in plants. *Curr. Sci.* 88 1771–1780.

[B36] Kaziro Y. Itoh H. Kozasa T. Nakafuku M. Satoh T. (1991). Structure and function of signal-transducing GTP-binding proteins. *Annu. Rev. Biochem.* 60 349–400 10.1146/annurev.bi.60.070191.002025 1909108

[B37] Kim M. D. Kim Y. H. Kwon S. Y. Jang B. Y. Lee S. Y. Yun D. J. (2011). Overexpression of 2-cysteine peroxiredoxin enhances tolerance to methyl viologen-mediated oxidative stress and high temperature in potato plants. *Plant Physiol. Biochem.* 49 891–897 10.1016/j.plaphy.2011.04.001 21620719

[B38] Kim S. T. Cho K. S. Jang Y. S. Kang K. Y. (2001). Two-dimensional electrophoretic analysis of rice proteins by polyethylene glycol fractionation for protein arrays. *Electrophoresis* 22 2103–2109 10.1002/1522-2683(200106)22:10<2103::AID-ELPS2103>3.0.CO;2-W 11465512

[B39] Knaupp M. Mishra K. B. Nedbal L. Heyer AG. (2011). Evidence for a role of raffinose in stabilizing photosystem II during freeze-thaw cycles. *Planta* 234 477–486 10.1007/s00425-011-1413-0 21533754

[B40] Koehler G. Wilson R. C. Goodpaster J. V. Sønsteby A. Lai X. Y. Witzmann F. A. (2012). Proteomic study of low-temperature responses in strawberry cultivars (*Fragaria* × ananassa) that differ in cold tolerance. *Plant Physiol.* 159 1787–1805 10.1104/pp.112.198267 22689892 PMC3425213

[B41] Kosmala A. Bocian A. Rapacz M. Jurczyk B. Zwierzykowski Z. (2009). Identification of leaf proteins differentially accumulated during cold acclimation between *Festuca pratensis* plants with distinct levels of frost tolerance. *J. Exp. Bot.* 60 3595–3609 10.1093/jxb/erp205 19553368

[B42] Kosová K. Vítámvás P. Planchon S. Renaut J. Vankova R. Prášil I. T. (2013). Proteome analysis of cold response in spring and winter wheat (*Triticum aestivum*) crowns reveals similarities in stress adaptation and differences in regulatory processes between the growth habits. *J. Proteome Res.* 18 4830–4845 10.1021/pr400600g 24047233

[B43] Kosová K. Vítámvás P. Prášil I. T. (2014). Proteomics of stress responses in wheat and barley – search for potential protein markers of stress tolerance. *Front. Plant Sci.* 5:711 10.3389/fpls.2014.00711 PMC4263075 25566285

[B44] Krishnan H. B. Natarajan S. S. (2009). A rapid method for depletion of RuBisCO from soybean (*Glycine max*) leaf for proteomic analysis of lower abundance proteins. *Phytochemistry* 70 1958–1964 10.1016/j.phytochem.2009.08.020 19766275

[B45] Lee D. G. Ahsan N. Lee S. H. Kang K. Y. Lee J. J. Lee B. H. (2007). An approach to identify cold-induced low-abundant proteins in rice leaf. *C. R. Biol.* 330 215–225 10.1016/j.crvi.2007.01.001 17434115

[B46] Lee D. G. Ahsan N. Lee S. H. Lee J. J. Bahk J. D. Kang K. Y. (2009). Chilling stress-induced proteomic changes in rice roots. *J. Plant Physiol.* 166 1–11 10.1016/j.jplph.2008.02.001 18433929

[B47] Liu H. M. Fang L. Che Y. S. Wu F. Z. Yang C. P. (2014). Protein expression patterns in two *Spiraea* species in response to cold treatment. *Mol. Biol. Rep.* 41 4533–4547 10.1007/s11033-014-3324-1 24639177

[B48] Parry M. A. J. Andralojc P. J. Mitchell R. A. C. Madgwick P. J. Keys A. J. (2003). Manipulation of RuBisCO: the amount, activity, function and regulation. *J. Exp. Bot.* 54 1321–1333 10.1093/jxb/erg141 12709478

[B49] Parsell D. A. Lindquist S. (1993). The function of heat-shock proteins in stress tolerance: degradation and reactivation of damaged proteins. *Annu. Rev. Genet.* 27 437–496 10.1146/annurev.ge.27.120193.002253 8122909

[B50] Portis A. R. (1990). RuBisCO activase. *Biochim. Biophys. Acta* 1015 15–28 10.1016/0005-2728(90)90211-L 2404515

[B51] Portis A. R. Li C. Wang D. Salvucci M. E. (2008). Regulation of RuBisCO activase and its interaction with RuBisCO. *J. Exp. Bot.* 59 1597–1604 10.1093/jxb/erm240 18048372

[B52] Rinalducci S. Egidi M. G. Karimzadeh G. Jazii F. R. Zolla L. (2011). Proteomic analysis of a spring wheat cultivar in response to prolonged cold stress. *Electrophoresis* 32 1807–1818 10.1002/elps.201000663 21710550

[B53] Sánchez-Bel P. Egea I. Sánchez-Ballesta M. T. Sevillano L. Del Carmen Bolarin M. Flores F. B. (2012a). Proteome changes in tomato fruits prior to visible symptoms. *Plant Cell Physiol.* 53 470–484 10.1093/pcp/pcr191 22227396

[B54] Sánchez-Bel P. Egea I. Sánchez-Ballesta M. T. Martinez-Madrid C. Fernandez-Garcia N. Romojaro F. (2012b). Understanding the mechanisms of chilling injury in bell pepper fruits using the proteomic approach. *J. Proteomics* 75 5463–5478 10.1016/j.jprot.2012.06.029 22796354

[B55] Sarvikas P. Hakala M. Pätsikkä E. Tyystjärvi T. Tyystjärvi E. (2006). Action spectrum of photoinhibition in leaves of wild type and npq1-2 and npq4-1 mutants of *Arabidopsis thaliana*. *Plant Cell Physiol.* 473 391–400 10.1093/pcp/pcj006 16415063

[B56] Schatz O. Oft M. Dascher C. Schebesta M. Rosorius O. Jaksche H. (1998). Interaction of the HIV-1 Rev cofactor eukaryotic initiation factor 5A with ribosomal protein L5. *Proc. Natl. Acad. Sci.U.S.A.* 95 1607–1612 10.1073/pnas.95.4.1607 9465063 PMC19115

[B57] Seki M. Satou M. Sakurai T. Akiyama K. Iida K. Ishida J. (2004). RIKEN *Arabidopsis* full length (RAFL) cDNA and its applications for expression profiling under abiotic stress conditions. *J. Exp. Bot.* 55 213–223 10.1093/jxb/erh007 14673034

[B58] Smith D. M. Stucker R. E. Elling L. J. (1979). “Fall dormancy in alfalfa: a valuable predictive tool,” in *Report of the 26th Improvement Conference. Agriculture Review and Manuals. Science and Education Administration*, (Saint Paul, MN: USDA-SEA), 34.

[B59] Sprague M. A. Fuelleman R. F. (1941). Measurements of recovery after cutting and fall dormancy of varieties and strains of alfalfa, *Medicago sativa*. *Agron. J.* 33 437–447 10.2134/agronj1941.00021962003300050006x

[B60] Spreitzer R. J. Salvucci M. E. (2002). RuBisCO: structure, regulatory interactions, and possibilities for a better enzyme. *Annu. Rev. Plant Biol.* 53 449–475 10.1146/annurev.arplant.53.100301.135233 12221984

[B61] Takahashi D. Kawamura Y. Uemura M. (2013). Changes of detergent-resistant plasma membrane proteins in oat and rye during cold acclimation: association with differential freezing tolerance. *J. Proteome Res.* 12 4998–5011 10.1021/pr400750g 24111712

[B62] Takahashi S. Kuzuyama T. Watanabe H. Seto H. (1998). A 1-deoxy-D-xylulose 5-phosphate reductoisomerase catalyzing the formation of 2-*C*-methyl-D-erythritol 4-phosphate in an alternative nonmevalonate pathway for terpenoid biosynthesis. *Proc. Natl. Acad. Sci. U.S.A.* 95 9879–9884 10.1073/pnas.95.17.9879 9707569 PMC21430

[B63] Thompson J. E. Hopkins M. T. Taylor C. Wang T. W. (2004). Regulation of senescence by eukaryotic translation initiation factor 5A: implications for plant growth and development. *Trends Plant Sci.* 9 174–179 10.1016/j.tplants.2004.02.008 15063867

[B64] Timmons F. L. Salmon S. C. (1932). The resistance of certain varieties and regional strains of alfalfa to controlled low temperatures. *Agron. J.* 24 642–655 10.2134/agronj1932.00021962002400080007x

[B65] Uváčková L’. Tak áčb T. Boehm N. Obert B. Šamaj J. (2012). Proteomic and biochemical analysis of maize anthers after cold pretreatment and induction of androgenesis reveals an important role of anti-oxidative enzymes. *J. Proteomics* 75 1886–1894 10.1016/j.jprot.2011.12.033 22252011

[B66] Vaclavik L. Mishra A. Mishra K. B. Hajslova J. (2013). Mass spectrometry-based metabolomic fingerprinting for screening cold tolerance in *Arabidopsis thaliana* accessions. *Anal. Bioanal. Chem.* 405 2671–2683 10.1007/s00216-012-6692-1 23325403

[B67] Veuthey A. L. Stucki J. (1987). The adenylate kinase reaction acts as a frequency filter towards fluctuations of ATP utilization in the cell. *Biophys. Chem.* 26 19–28 10.1016/0301-4622(87)80003-0 3036264

[B68] Vítamìvasì P. Prasíil I. T. Kosová K. Planchon S. Renaut J. (2012). Analysis of proteome and frost tolerance in chromosome 5A and 5B reciprocal substitution lines between two winter wheats during long-term cold acclimation. *Proteomics* 12 68–85 10.1002/pmic.201000779 22065556

[B69] Voet D. Voet J. G. (1995). *Biochemistry.* New York: John Wiley & Sons, Inc. 10.1002/bmb.20 21591048

[B70] Whitney S. M. Andrews T. J. (2001). Plastome-encoded bacterial ribulose-1,5-bisphosphate carboxylase/oxygenase (RuBisCO) supports photosynthesis and growth in tobacco. *Proc. Natl. Acad. Sci. U.S.A.* 98 14738–14743 10.1073/pnas.261417298 11724961 PMC64751

[B71] Xi J. Wang X. Li S. Zhou X. Yue L. Fan J. (2006). Polyethylene glycol fractionation improved detection of low-abundant proteins by two-dimensional electrophoresis analysis of plant proteome. *Phytochemistry* 67 2341–2348 10.1016/j.phytochem.2006.08.005 16973185

[B72] Xu J. Li Y. Sun J. Du L. Zhang Y. Yu Q. (2013). Comparative physiological and proteomic response to abrupt low temperature stress between two winter wheat cultivars differing in low temperature tolerance. *Plant Biol.* 15 292–303 10.1111/j.1438-8677.2012.00639.x 22963252

[B73] Yan S. P. Zhang Q. Y. Tang Z. C. Su W. A. Sun W. N. (2006). Comparative proteomic analysis provides new insights into chilling stress responses in rice. *Mol. Cell. Proteomics* 5 484–496 10.1074/mcp.M500251-MCP200 16316980

[B74] Zeleznikar R. J. Dzeja P. P. Goldberg N. D. (1995). Adenylate kinase-eatalyzed phosphoryl transfer couples ATP utilization with its generation by glycolysis in intact muscle. *J. Biol. Chem.* 270 7311–7319 10.1074/jbc.270.13.7311 7706272

[B75] Zhang S. Feng L. H. Jiang H. Ma W. J. Korpelainen H. Li C. Y. (2012). Biochemical and proteomic analyses reveal that *Populus cathayana* males and females have different metabolic activities under chilling stress. *J. Proteome Res.* 11 5815–5826 10.1021/pr3005953 23072643

[B76] Zhou R. Jackson L. Shadle G. Nakashima J. Temple S. Chen F. (2010). Distinct cinnamoyl CoA reductases involved in parallel routes to lignin in *Medicago truncatula*. *Proc. Natl. Acad. Sci. U.S.A.* 107 17803–17808 10.1073/pnas.1012900107 20876124 PMC2955080

[B77] Zuther E. Schulz E. Childs L. H. Hinch D. K. (2012). Clinal variation in the non-acclimated and cold-acclimated freezing tolerance of *Arabidopsis thaliana* accessions. *Plant Cell Environ.* 35 1860–1878 10.1111/j.1365-3040.2012.02522 22512351

